# Surface Acoustic Wave Device with Reduced Insertion Loss by Electrospinning P(VDF–TrFE)/ZnO Nanocomposites

**DOI:** 10.1007/s40820-016-0088-2

**Published:** 2016-03-17

**Authors:** Robin Augustine, Frederic Sarry, Nandakumar Kalarikkal, Sabu Thomas, Laurent Badie, Didier Rouxel

**Affiliations:** 1 International and Inter University Centre for Nanoscience and Nanotechnology, Mahatma Gandhi University, Kottayam, Kerala 686 560 India; 2 School of Pure and Applied Physics, Mahatma Gandhi University, Kottayam, Kerala 686 560 India; 3 School of Chemical Sciences, Mahatma Gandhi University, Kottayam, Kerala 686 560 India; 4 Institut Jean Lamour, Université de Lorraine, 54000 Vandoeuvre-Lès Nancy, France; 5 CNRS, IJL UMR 7198, 54000 Vandoeuvre-Lès Nancy, France; 6 Department of Materials Science and Engineering, Technion-Israel Institute of Technology, De-Jur Building, Technion City, 3200003 Haifa, Israel

**Keywords:** Surface acoustic wave, SAW, P(VDF−TrFE), ZnO, Biosensor

## Abstract

Surface acoustic wave (SAW) devices have been utilized for the sensing of chemical and biological phenomena in microscale for the past few decades. In this study, SAW device was fabricated by electrospinning poly(vinylidenefluoride-co-trifluoroethylene) (P(VDF−TrFE)) incorporated with zinc oxide (ZnO) nanoparticles over the delay line area of the SAW device. The morphology, composition, and crystallinity of P(VDF−TrFE)/ZnO nanocomposites were investigated. After measurement of SAW frequency response, it was found that the insertion loss of the SAW devices incorporated with ZnO nanoparticles was much less than that of the neat polymer-deposited device. The fabricated device was expected to be used in acoustic biosensors to detect and quantify the cell proliferation in cell culture systems.

## Introduction

For the past few decades, there has been an increased attention for the development of surface acoustic wave (SAW) devices for various applications such as electronic components, microfluidic actuators, and especially sensors [1, 2]. These devices are interesting because of their tunability to make it compatible with the frequencies of today’s electronic devices. The major advantages of using a SAW transducer are its high sensitivity and cost effectiveness [3]. SAW devices based on lithium niobate (LiNbO_3_) crystals in particular have long been known for their high acoustic wave generation [4]. The sensor principle is based on the modification of the oscillation frequency of the surface acoustic wave when the surface is subjected to physical or chemical perturbations.

Recent attempts have been focused on the development of nanostructured coatings on SAW devices to improve the sensitivity of the sensors, for instance for gas or humidity detection [5, 6]. A more surface-confined acoustic wave can be obtained by depositing guiding layers over the delay line area of the SAW device. Both the variation in electrical conductivity and mass of the layer disturb the velocity of SAW due to mechanical and piezoelectric effects.

Recent reports suggested that depositing nanostructured coatings on SAW via electrospray or electrospinning techniques improves the electrical response of the SAW device [7]. Electrospinning can produce continuous nanofibers from submicron diameter scale down to nanometer diameter scale through an electrically charged jet of polymer solution [8–13]. Large surface area-to-volume ratio and high porosity make electrospun membranes highly promising for the development of ultrasensitive biosensing devices [14].

The major goal of this study is to fabricate an electrospun piezoelectric polymer-based scaffold [15, 16] with the high sensitivity of SAW device in order to follow bio-reactions taking place in the scaffold when it is used in an in vitro cell culture system. This deposition allows a direct nano-patterning on the delay line of the SAW device. It can help monitor sharply the dynamism of the cell–scaffold reaction involved and provide a tool for better understanding of cell proliferation in a cell culture system. To develop such device, a first step presented in this paper is to characterize the influence of the polymer deposition on the performance of the SAW device.

For the electrospun scaffold, the polymeric material chosen in this study is a piezoelectric poly(vinylidenefluoride-co-trifluoroethylene) (P(VDF–TrFE)). PVDF and its copolymer P(VDF–TrFE) have been exploited in wide applications due to their ferroelectricity, piezoelectricity, and pyroelectricity [17–19]. P(VDF–TrFE) copolymers have been in particular used for sensing and actuation [20], even if their piezoelectricity (as measured by their piezoelectric coefficients) is significantly lower than that of highly piezoelectric materials like lead zirconate titanate (Pb[Zr_x_Ti_1-x_]O_3_). A major advantage of PVDF and P(VDF–TrFE) copolymer over conventional rigid piezoelectric materials is their high flexibility that makes them ideal candidates for instrumentation where a thin coating is required over structural surfaces. The ferroelectricity of P(VDF–TrFE) makes it also a good candidate for acoustic devices [21]. P(VDF–TrFE) has a higher electromechanical (EM) coupling factor than PVDF and also higher acoustical impedance to maximize energy transfer between fibers and substrate [22, 23]. Moreover, according to the previous reports, electrospun membranes do not require to be polarized in order to achieve the desired piezoelectric properties because the poling is naturally done by the electrical field applied during electrospinning [23]. However, their relatively low piezoelectricity inspires and stimulates the development of PVDF and P(VDF–TrFE) nanocomposites with improved piezoelectricity and additional or tunable mechanical, dielectric, or optical properties, with various nanofillers like for instance aluminum oxide (Al_2_O_3_) [24], lithium niobate (LiNbO_3_) [25], or ZnO [26].

ZnO is well known for its good piezoelectric properties, high electromechanical coupling coefficient, high-temperature stability, and potential to be integrated with surface acoustic wave devices [27]. As thin-film coating, ZnO nanoparticles may themselves be used as piezoelectric substrates for SAW device [28]. ZnO nanostructures on LiNbO_3_ transducers have been used for gas and biosensing applications [29, 30]. Polymer nanocomposites containing ZnO nanoparticles are reported for biomedical applications due to their relatively good biocompatibility [31–34]. ZnO nanostructures show both semiconducting and piezoelectric properties [35]. These two properties together make them very promising candidates for the fabrication of acoustic wave devices for various applications [28, 36].

Both P(VDF–TrFE) [37] and ZnO nanoparticles [31–33] are relatively biocompatible and are known to promote cell adhesion and proliferation, therefore this device may have a high potential to be further developed as a biosensing system to monitor the cell proliferation in in vitro cell culture systems. Incorporation of ZnO nanoparticles in electrospun P(VDF–TrFE) membranes may also impart superior piezoelectric response to the P(VDF–TrFE) nanocomposite while maintaining its excellent flexibility, chemical stability, and adaptability to the irregular surfaces. Large surface area-to-volume ratio as well as porous nature of electrospun membranes can achieve much better sensitivity due to higher contact area for the attachment of cells. The deposition of P(VDF–TrFE)/ZnO nanocomposite fibers on the SAW device may improve the electrical response. In this paper, we report the fabrication and characterization of electrospun P(VDF–TrFE)/ZnO nanocomposites deposited on LiNbO_3_ SAW device and the acoustic response upon stimulation.

## Experimental

### Fabrication of SAW Device

**Fig. 1:**
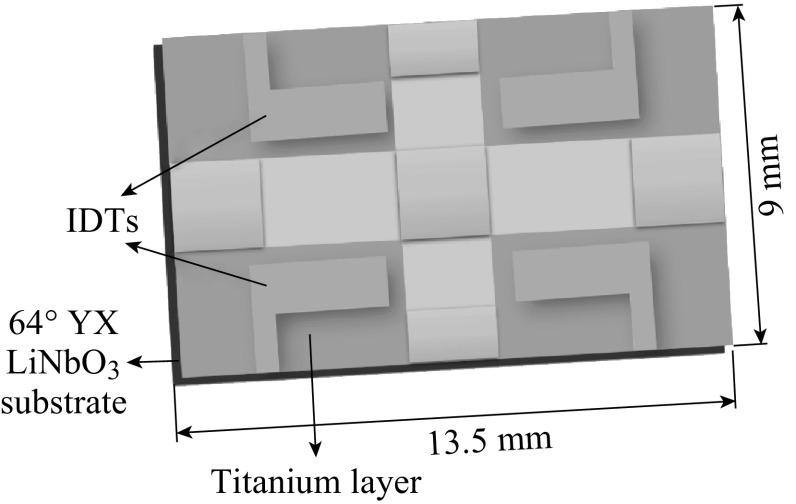
Schematic representation of the SAW device which was used for the deposition of electrospun P(VDF–TrFE)/ZnO nanocomposites

The SAW transducer pattern was made on a 64° YX LiNbO_3_ substrate as reported by Sadek et al. [38] with slight modifications. A shear horizontal (SH) leaky surface acoustic wave is the major mode in this substrate. A schematic representation of the SAW device is shown in Fig. 1. The transducer consists of a two-port resonator with 38 electrode pairs in both input and output inter-digital transducers (IDTs). It also has 160 electrodes, 700 μm aperture width, and a periodicity of 40 μm. Further, a two-port resonator structure was selected over the delay line as its higher phase slope increases oscillation stability. The IDTs were fabricated by patterning an 80-nm gold (Au) layer. The Au layer was deposited upon 20 nm titanium (Ti) for improved adhesion to the substrate.

### Electrospinning of P(VDF–TrFE) on SAW Device

The electrospinning apparatus consists of a syringe pump, a high voltage power supply, and a 10-mL syringe (attached with a 21G diameter needle). A rotating mandrel with a rotation speed of 1000 rpm was used as the collector. The needle-to-collector distance was maintained at 10 cm with an applied voltage of 18 kV. The feeding rate of the solution was precisely controlled by a syringe pumping system which was adjusted to a flow rate of 1.5 mL h^−1^.

**Fig. 2:**
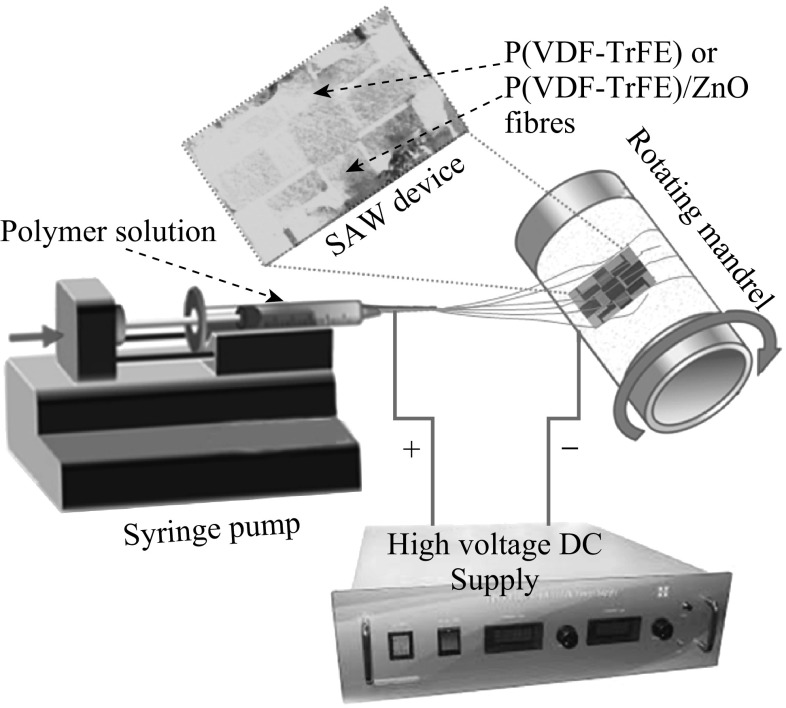
Schematic representation of the electrospinning process of P(VDF–TrFE) and P(VDF–TrFE)/ZnO nanocomposites over the SAW device

The overall synthetic procedure is illustrated in Fig. 2. ZnO nanoparticles (NanoGard^®^, Alfa Aesar) were dispersed in acetone by ultrasonication. Then, the required weight percentage of P(VDF–TrFE) 60/40 (M_w_ ~ 500,000 g mol^−1^, provided by Piezotech SAS of France) was dissolved in the above solution until the polymer gets dissolved. Proper ultrasonication condition is essential to obtain the optimum dispersion and homogeneity of the final material [39]. 14 wt% of P(VDF–TrFE)/ZnO solution containing various weight percentages of ZnO nanoparticles was electrospun at optimized electrospinning condition as mentioned above. To get a thin layer of electrospun fibers over the SAW device, electrospinning process was carried out for 5 min. Finally, fibers deposited on unwanted areas of the SAW device were carefully removed using acetone.

### Characterization

#### Scanning Electron Microscopy (SEM)

The morphology of the deposited fiber membranes over SAW devices was observed by SEM. The membranes were peeled out from the SAW device and carefully sectioned with an approximate size of 3 mm length and 0.5 mm width using a sharp scissor and mounted on an SEM sample holder. Before mounting in the microscope, each sample was coated with platinum. A Philips XL-30 FEG scanning electron microscope at 5 kV was used to analyze the samples.

#### Energy-Dispersive X-ray Spectroscopy (EDX)

The presence of ZnO nanoparticles in the deposited P(VDF–TrFE) fiber membrane was confirmed by EDX analysis using Philips XL-30 FEG SEM with EDS (EDAX), based on the energy and intensity distribution of X-ray signals generated by the electron beam striking the surface of the specimen.

#### Fourier Transform Infrared Spectroscopy (FTIR)

Electrospun P(VDF–TrFE) membranes and P(VDF–TrFE)/ZnO nanocomposite membranes which were peeled out from the SAW device were subjected to IR analysis. The FTIR spectra were collected over a range of 500–4000 cm^−1^ with a Perkin Elmer Spectrum 400 FTIR spectrometer with PIKE Gladi ATR (attenuated total reflectance) attachment and DTGS detector on a diamond crystal with 15 scans at 4 cm^−1^ resolution using Spectrum 400 software 62 (version 6.3). Since the ATR was used for the measurement, semi-quantitative information regarding the relative amount of various crystalline phases can be obtained.

#### Differential Scanning Calorimetry (DSC)

Crystallinity is an important characteristic property of polymers that determines the physical properties of any polymer like mechanical stability and degradation. It can also give important information regarding the crystalline phases of P(VDF–TrFE) and its copolymers. Crystallization and melting of the nanocomposites were investigated using a TA Instruments Q200 DSC. P(VDF–TrFE) has a glass transition temperature (*T*_g_) of 30–40 °C, melting (*T*_m_) temperature of 140–195 °C, and crystallization temperature (*T*_c_) of 100–165 °C, depending on the crystalline nature of the polymer. Measurements were carried out under a nitrogen flow of 20 mL min^−1^. The samples were heated from −60 to 200 °C at 10 °C min^−1^. The samples were kept for 1 min at 80 °C to eliminate the thermal history and then cooled at 10 °C min^−1^ to −200 °C.

#### SAW Frequency Response Measurements

**Fig. 3:**
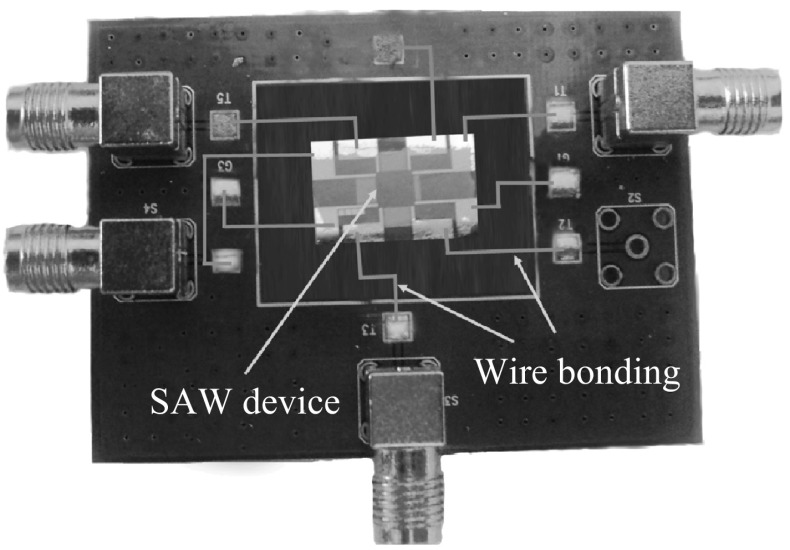
Photograph of a SAW device with electrospun P(VDF–TrFE)/ZnO nanocomposites connected on a PCB

The fabricated neat P(VDF–TrFE) membranes as well as P(VDF–TrFE)/ZnO nanocomposite membranes containing 1, 2, and 4 wt% ZnO nanoparticles were attached on printed circuit boards (PCBs) having two inputs and two outputs. The IDTs of the SAW devices were connected to the ports of the PCB by silver paste bonding on the SAW device and wire bonding on the PCB as shown in Fig. 3.

The experimental frequency characterization of the IDT response was performed using an E5061B Agilent Network Analyzer. The SAW response was measured in terms of insertion loss of the S21 transmission coefficient parameter. The center frequency *f*_i_ of each peak is given by the formula: *f*_i_ = *v*_i_/*l*, where *v*_i_ is the acoustic velocity depending on the different layers and *l* is the wavelength fixed by the spatial periodicity of the IDT. The sensor consists of a transmission line which transmits a mechanical signal in the form of an acoustic wave launched by the input port (input IDT) due to the applied RF electrical signal. After a particular time delay, the traveling acoustic wave will be converted back to an electric signal in the output port. Changes in the coating layer and/or in the semi-infinite fluid medium can produce variations in the acoustic wave properties. These variations can be measured comparing the input and output electrical signals, since *V*_in_ remains unchanged, while *V*_out_ changes. Thus, from an electric point of view, the delay line is determined by its transfer function *H*(*f*) = *V*_out_/*V*_in_, which represents the relationship between input and output electrical signals. Thus, *H*(*f*) is a complex number that can be defined as *H*(*f*) = *A*e^jφ^, where *A* is the amplitude, i.e., *A* = |*V*_out_/*V*_in_|, and φ the phase-shift between *V*_out_ and *V*_in_. The insertion loss (IL) in dB is given by 20log*A*.

## Results and Discussion

### Morphologies of P(VDF–TrFE)/ZnO Nanocomposites

**Fig. 4:**
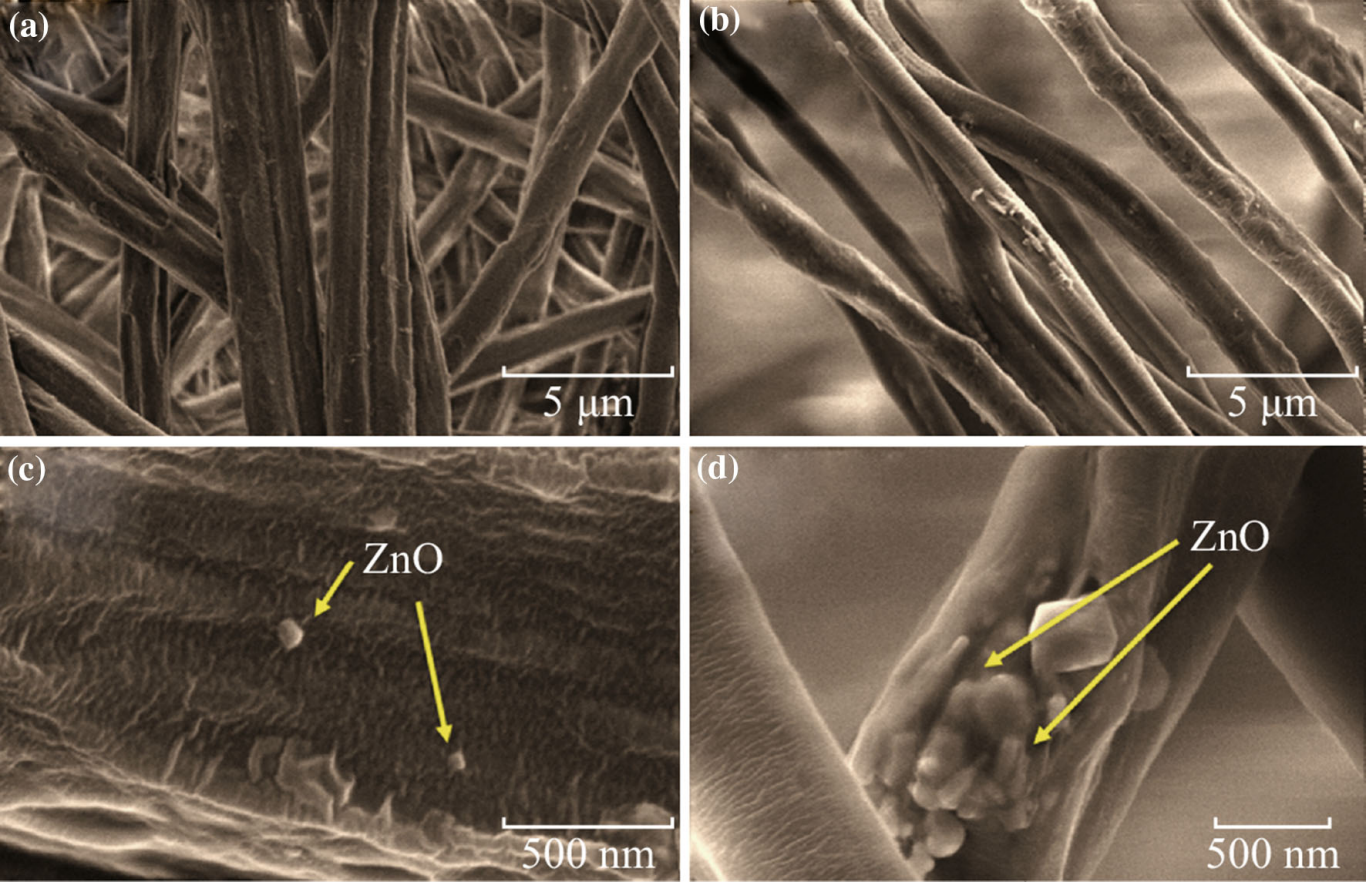
SEM images of electrospun P(VDF–TrFE) nanocomposites with **a** 1 wt% ZnO nanoparticles and **b** 4 wt% ZnO nanoparticles; **c** higher magnification image of (**a**); **d** higher magnification image of (**b**), showing the presence of ZnO nanoparticles on fibers

Morphologies of P(VDF–TrFE) membranes with different concentrations of ZnO nanoparticles which were electrospun on the SAW device are shown in Fig. 4 (4a and 4c for 1 wt% nanocomposite, and 4b and 4d for 4 wt% nanocomposite). All the deposited fibers have uniform diameters and there is no significant difference in the two concentrations (see Fig. 4a, b). Higher magnification SEM micrographs confirm the presence of well-dispersed ZnO nanoparticles on the fibers at lower concentrations of ZnO nanoparticles (Fig. 4c), whereas ZnO nanoparticles are agglomerated at higher concentration (Fig. 4d).

### Composition of P(VDF–TrFE)/ZnO Nanocomposites

**Fig. 5:**
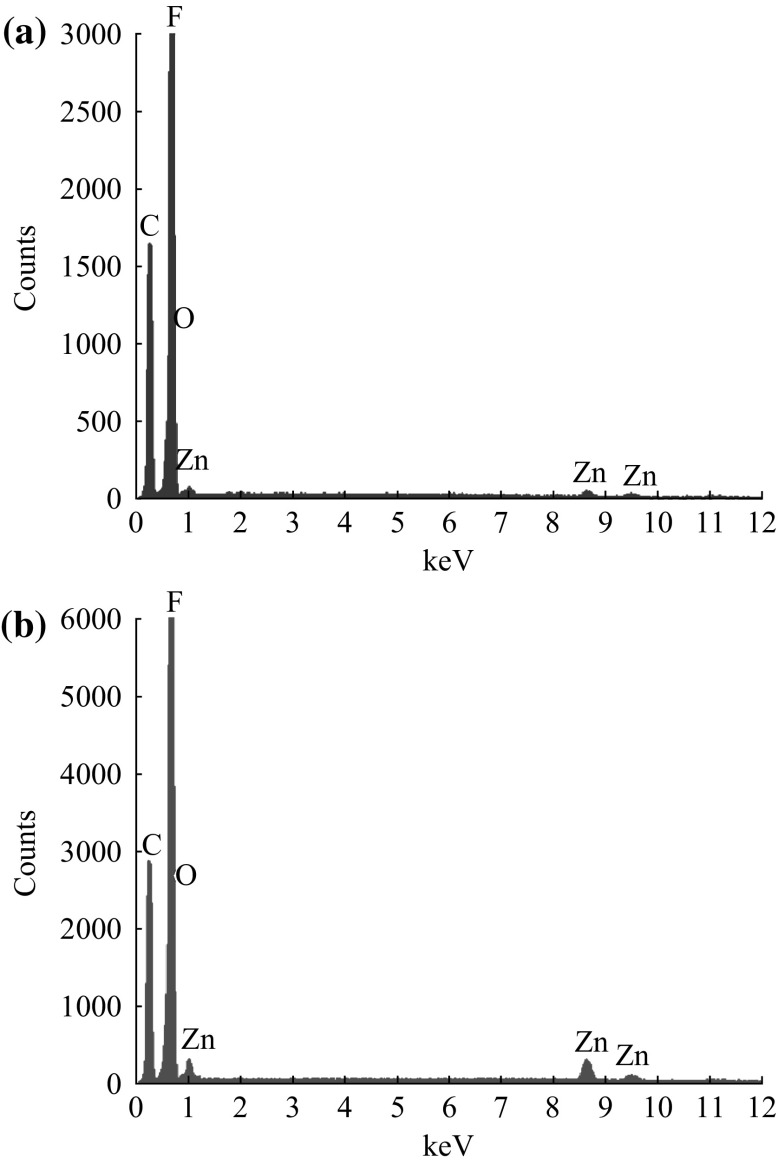
Representative EDX spectrum of P(VDF–TrFE) nanocomposites containing **a** 1 wt% and **b** 4 wt% ZnO nanoparticles electrospun on SAW devices

Representative EDX spectra of P(VDF–TrFE) membranes that are incorporated with 1 and 4 wt% ZnO nanoparticles are shown in Fig. 5. There are some sharp low-energy peaks corresponding to the elements carbon (*K*_*α*_ radiation with 0.277 keV) and fluorine (*Kα* radiation with 0.677 keV) of the P(VDF–TrFE) which were present in the EDX spectra of all the P(VDF–TrFE)/ZnO nanocomposites as well as neat P(VDF–TrFE) membranes. In the case of ZnO nanoparticle-incorporated P(VDF–TrFE) nanocomposite membranes, three additional peaks were observed at the energy levels 1.01 keV (*L*_*α*_), 8.63 keV (*K*_*α*_), and 9.5 keV (*Kβ*) which are the characteristic of the zinc element. From the spectra, it was difficult to visually distinguish the oxygen of ZnO nanoparticles due to the fact that both oxygen and fluorine are the nearest elements in the periodic table with k*α* emissions of 0.525 and 0.677, respectively.

### ATR-FTIR Analysis

**Fig. 6:**
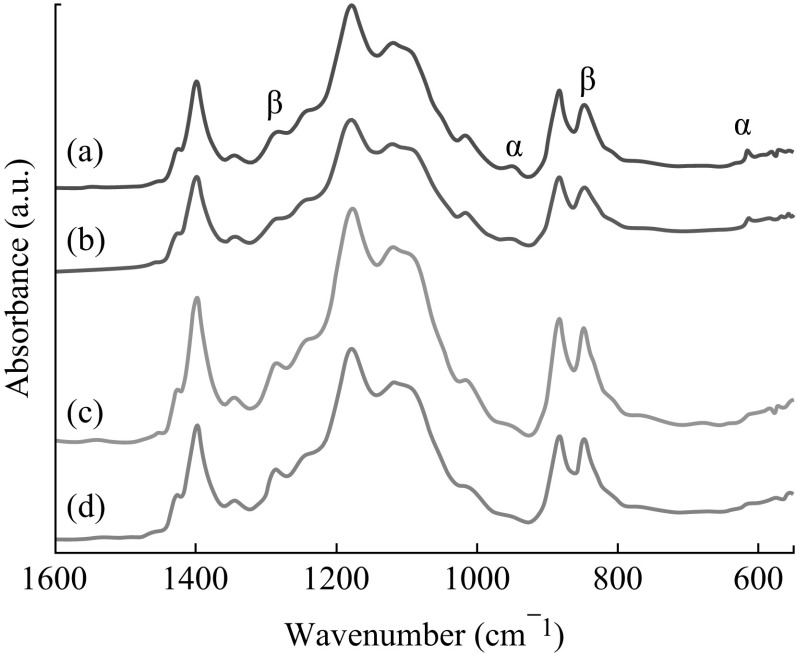
ATR-FTIR spectra of **a** neat P(VDF–TrFE), and P(VDF–TrFE)/ZnO nanocomposites with **b** 1 wt%, **c** 2 wt%, and **d** 4 wt% of ZnO nanoparticle content which were deposited on SAW device

ATR-FTIR spectra of electrospun bare P(VDF–TrFE) and P(VDF–TrFE)/ZnO nanocomposite membranes with varying concentrations of ZnO nanoparticles are shown in Fig. 6. The observed patterns originate from oscillations of large parts of the polymer chain skeleton and/or the skeleton and attached functional groups. The vibrational modes of the polymer chains in P(VDF–TrFE) can be used to distinguish the different phases present in this polymer. Most infrared-active vibrations for the copolymer are concentrated in a narrow region between 1500 and 600 cm^−1^. Very weak peaks at 974 and 615 cm^−1^ in neat P(VDF–TrFE) membranes are due to the non-polar *α* phase [40], whereas the characteristic peaks at 1285 and 847 cm^−1^ correspond to the electroactive *β* phase [41, 42]. The peaks 1455, 1430, 1385, 1212, 1152, 854, 796, and 1385 cm^−1^ corresponding to *α* phase were completely absent in the spectra. Incorporation of ZnO nanoparticles leads to a significant increase in the presence of the *β* phase, whereas the *α* phase gets diminished as evident from the intense vibrational bands corresponding to *β* phase and the reduced bands corresponding to *α* phase, respectively. The presence of the ZnO nanoparticles may contribute to the piezoelectricity of P(VDF–TrFE) nanocomposite membranes due to the increase in *β* phase [43]. When the nanoparticle concentration increased to 2 or 4 wt%, the intensity of peaks corresponding to *β* phase was significantly increased. It was very prominent in the case of 1285 and 847 cm^−1^ peak. There was a sharp decrease in the intensity of the peaks corresponding to the *α* phase of the polymer, which were present at 974 and 615 cm^−1^ when the ZnO nanoparticle concentration increased in the polymer matrix.

### DSC Analysis

**Fig. 7:**
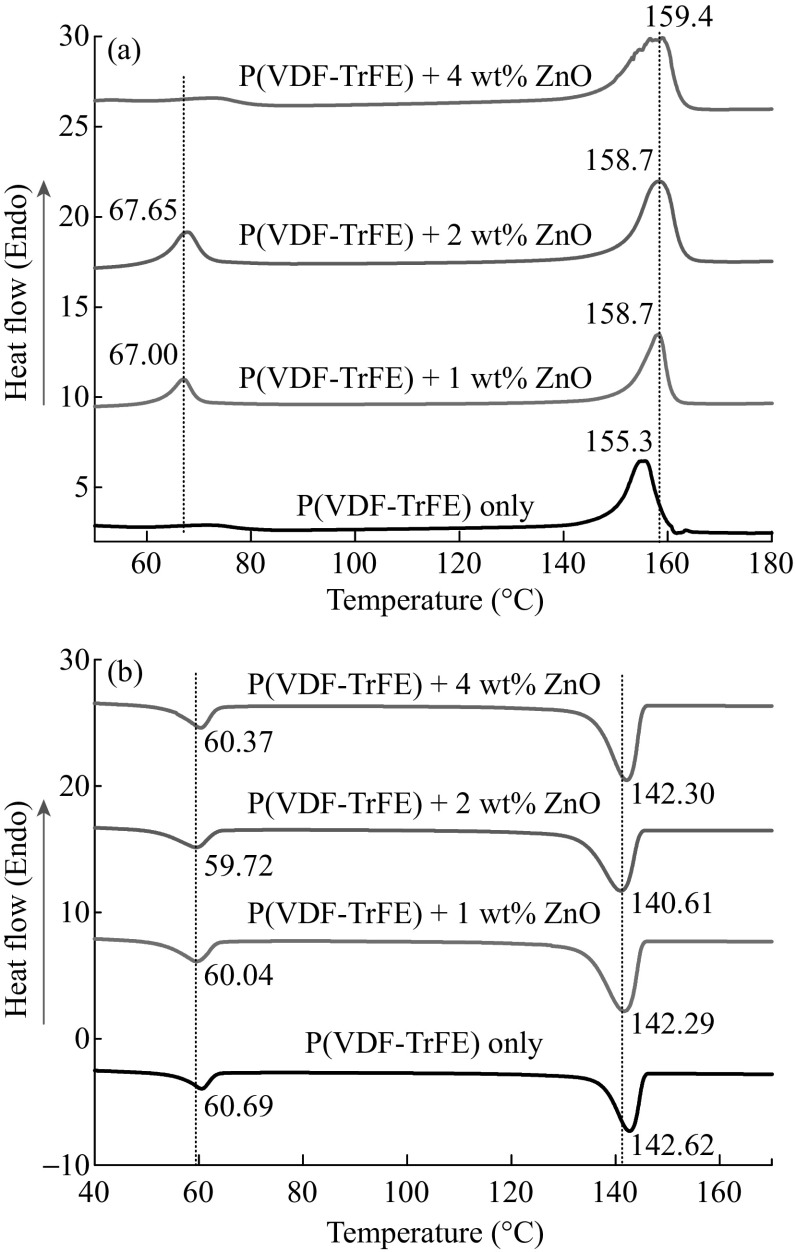
Endothermic transition peaks **a** during the melting of P(VDF–TrFE) and its nanocomposites with ZnO, and **b** during the crystallization of poly(VDF–TrFE) and its nanocomposites with ZnO

DSC thermograms of the deposited electrospun P(VDF–TrFE) membranes and the P(VDF–TrFE)/ZnO nanocomposite membranes showed some variation in endothermic and exothermic peaks. Figure 7 presents typical heating and cooling DSC thermograms for the P(VDF–TrFE) with various ZnO nanoparticle concentrations. During heating (Fig. 7a), two endothermic regions were observed for all the fabricated membranes. The first peak at around 67 °C corresponds to the ferroelectric-to-paraelectric transition (Curie temperature, *T*_C_). The second transition at around 158 °C is related to the melting of the crystalline phase (*T*_m_). For the neat P(VDF–TrFE) *T*_C_ is ill-defined. Instead of an expected sharp peak, a broad less intense peak was observed in between 60 and 80 °C. While incorporating ZnO nanoparticles in the polymer matrix, the melting peak as well as Curie temperature shifts to a higher temperature region. This is due to the fact that the incorporation of ZnO nanoparticles results in the increase in size of crystallites in the copolymer. The low-temperature Curie transition takes place in less ordered crystalline phases, whereas high-temperature Curie transitions are attributed to well-formed crystallites [44]. However, 4 wt% ZnO nanoparticles lead to a decrease in the peak intensity corresponding to the *T*_C_. Higher filler loading above 4 wt% resulted in nanofiller agglomerates which hinder the nucleation and thinner lamellar crystals were formed due to this disruption in the crystallization process. Previous report suggests that the nucleation rate increases as an inverse exponential power of filler size [45].

The broad melting endothermic peak can be described as a superposition of the melting peaks for the lower melting *α* phase and the higher melting *β* phase. While incorporating a low concentration of ZnO nanoparticles, the endothermic peaks shift toward higher values. This endothermic peak is weighted toward higher temperatures, which is indicative of the relative amount of *β* phase to *α* phase in these samples [46, 47].

During the cooling process, exothermic peaks appear at crystallization temperature (*T*_c_) and paraelectric-to-ferroelectric transition temperature (*T*_p–f_) (Fig. 7b). The neat P(VDF–TrFE) membranes exhibited a crystallization and para–ferroelectric (p–f) transition at around 142 and 60 °C, respectively. These are comparable with the reported results for P(VDF–TrFE) copolymers [30]. At a lower content of ZnO nanoparticles, there was a decrease in the para–ferroelectric transition temperature as well as the crystallization temperature. This was due to the nucleation effect of ZnO nanoparticles in the P(VDF–TrFE) polymer matrix. ZnO nanoparticles initiated the crystallization at lower temperature than the neat P(VDF–TrFE). However, there was a slight increase in both these transition temperatures at 4 wt% of ZnO nanoparticles. This increase might be due to the agglomerates of ZnO nanoparticles that interrupted the molecular motion of the polymer chains at lower temperatures to form crystallites.

### SAW Frequency Response Measurements

**Fig. 8:**
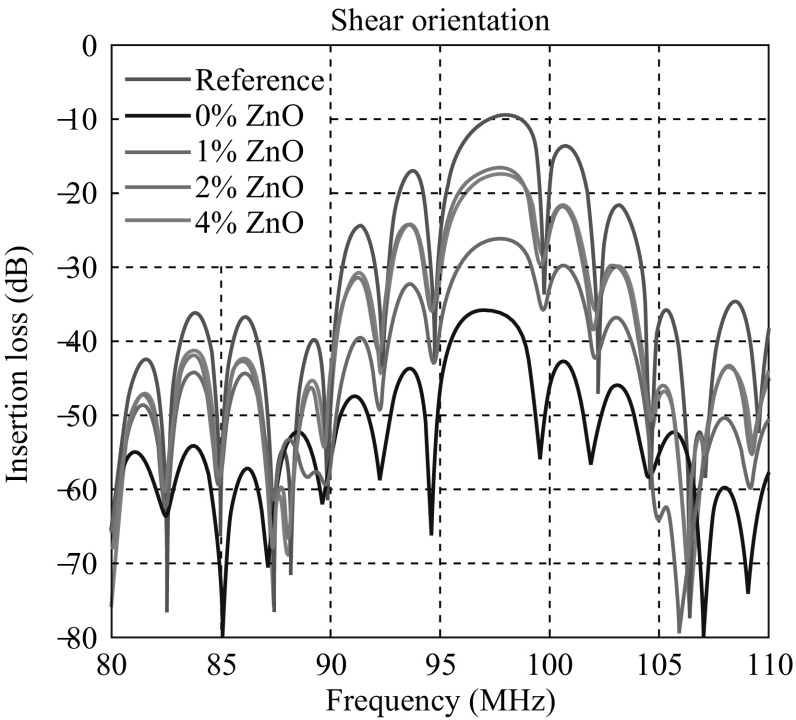
Shear responses observed for the different samples in the frequency region of 80–110 MHz

From the frequency response characteristics of the SAW devices deposited with neat P(VDF–TrFE) and that containing various concentrations of ZnO nanoparticles, the corresponding insertion losses were measured and compared. The devices were fabricated on 128 Y–X LiNbO_3_ substrates with the electrode designed to generate a shear wave so that the energy will not be absorbed by the polymer deposited on the wave path. Frequency response characteristics of the fabricated devices are shown in Fig. 8. The frequency responses were observed in the range of 80–110 MHz. The SAW device without any polymer deposition (reference sample) had a center frequency of 98 MHz and had shown an insertion loss of 9.52 dB. Deposition of electrospun neat P(VDF–TrFE) over the delay line area of the SAW device leads to a considerable decrease in the device performance in terms of insertion loss. It showed 35.9 dB insertion loss at 96.9 MHz.

The SAW delay lines deposited with P(VDF–TrFE) containing 1 wt% ZnO nanoparticles had a center frequency of 97.8 MHz and showed an insertion loss of 26.1 dB. Insertion loss decreased as the concentration of ZnO nanoparticles increased in the P(VDF–TrFE) polymer matrix. Incorporation of 2 wt% of ZnO nanoparticles further substantially reduced the insertion loss to 17.4 dB at 97.8 MHz. With the addition of 4 wt% ZnO nanoparticles, the additional reduction of the insertion loss was marginal (16.6 dB at 97.6 MHz). However, 2 wt% was sufficient enough to reach exploitable response of the device. This composition might probably be the best in order to maintain the flexibility of the material while maintaining appreciable device performance.

The device parameters of SAW devices were found to be significantly improved by the deposition of electrospun P(VDF–TrFE)/ZnO nanocomposite membranes on the delay line area. In particular, among all the SAW devices considered in this work, the devices deposited with electrospun P(VDF–TrFE) containing 2 and 4 wt% ZnO nanoparticles have the best device performance. ZnO nanoparticles are endowed with large electromechanical coupling coefficient [45]. The presence of ZnO nanoparticles in the polymer matrix may enhance the electromechanical coupling coefficient of the deposited layer [28]. Further, FTIR analysis demonstrated that there was an increase in the piezoelectric crystalline phase of P(VDF–TrFE) in the polymer matrix when ZnO nanoparticles are incorporated. This might be the major reason of the reduction in insertion loss for the SAW devices deposited with P(VDF–TrFE)/ZnO nanocomposites.

## Conclusion

In summary, SAW sensor device was fabricated by electrospinning P(VDF–TrFE)/ZnO nanocomposites over the delay line area of the SAW device. Incorporation of ZnO nanoparticles in the polymer matrix enhanced the formation of *β* phase in the copolymer. When incorporating 1 and 2 wt% of ZnO nanoparticles in the P(VDF–TrFE), the insertion loss for the SAW device was much less than that of neat polymer-deposited device. The fabricated device was promising to be used as a scaffold for cell attachment in in vitro cell culture systems to monitor or quantify cells.
